# Unexpected Casualty: The Accidental Fatality of a Glass Injury

**DOI:** 10.7759/cureus.80797

**Published:** 2025-03-18

**Authors:** Toshal Wankhade, Ashok Kumar Rastogi, Abhishek Parashar, Amit Patil, Chaitanya Mittal

**Affiliations:** 1 Forensic Medicine and Toxicology, All India Institute of Medical Sciences, Patna, Patna, IND

**Keywords:** accidental glass injury, blood vessels tear, circumstantial evidence, crime scene evidence, manner of death, medicolegal autopsy, sharp fatal glass wound

## Abstract

Fatal sharp-cut injuries are often associated with violent acts and are typically considered homicidal in nature. This case report presents a rare instance of an accidental fatality caused by glass, initially suspected as a homicide due to the injury pattern and preliminary crime scene scenario. It describes a 20-year-old male found dead on the roadside with a deep, wide, sharp wound to his right elbow and significant blood loss. An autopsy confirmed hemorrhagic shock due to a major vessel rupture. Further investigation, including CCTV footage, revealed that the intoxicated victim had accidentally struck a broken glass door. This case discusses the characteristics of glass-induced injuries and underscores the importance of integrating forensic analysis with investigative findings to prevent misclassification of the manner of death.

## Introduction

Injuries caused by pointed objects or sharp-edged objects are known as sharp-force injuries. Fatal sharp injuries, particularly those involving deep cuts, are frequently assumed to be homicidal in manner [1]. Accidental sharp force fatalities may occur but are relatively rare in occurrence [2]. The current case is about a fatal sharp force wound that occurred accidentally. In this case, a sharp, fatal wound was produced by a glass particle on the flexor aspect of the right elbow, resembling a chopped wound due to its dimensions. Such kinds of injuries are often associated with violent acts, typically homicide [3]. The same confusion had occurred in this case also; the case was initially assumed to be homicidal due to the nature of the injury and the allegation during the early inquiry. However, upon further investigation, the case was proved to be an accidental injury caused by a sharp blow from broken glass.

Injuries caused by glass are not uncommon in occurrence and can range from minor cuts to severe, life-threatening wounds. Like in the case of a vehicular accident, when toughened windscreen glass breaks into small dice-shaped fragments, it produces the characteristic small cuts known as ‘sparrow foot’ marks on the body. Glass injuries may result in lacerations, incised wounds, or stab wounds depending on the nature of the glass fragment striking the body [4]. Injury by glass may result in a fatal sharp wound, but their occurrence is not common, and cases of sharp fatality due to glass are usually reported in the form of an accident [3,5]. Therefore, this case serves as a reminder that fatal sharp injuries can result from glass particles in accidental circumstances.

This case also highlights the complexities involved in determining the manner of death, which remains a significant challenge in medicolegal autopsies. Determining the manner of death, i.e., whether the case is homicidal, suicidal, or accidental, is one of the difficult decisions for forensic pathologists and legal authorities. Though determining the manner of death is not a legal function of a forensic pathologist, his experience and training always help legal officials make decisions regarding the classification of the manner of death [4].

In clinical practice, history plays a pivotal role in diagnosing diseases. However, in forensic practice, reliance on history alone can be misleading, as the deceased are not here to provide their narration, and incomplete investigations may yield inaccurate information [6]. The manner of death is a critical component of medicolegal certification, but it mostly relies on the circumstances surrounding the death. While autopsy findings provide valuable insights, they have limitations and must be corroborated with circumstantial evidence [4]. In cases where the manner of death is unclear, it is essential to document it as "pending investigation" to avoid premature conclusions [7]. This case also underscores the importance of circumstantial evidence in forensic investigations, such as CCTV footage.

## Case presentation

History

**Figure 1:**
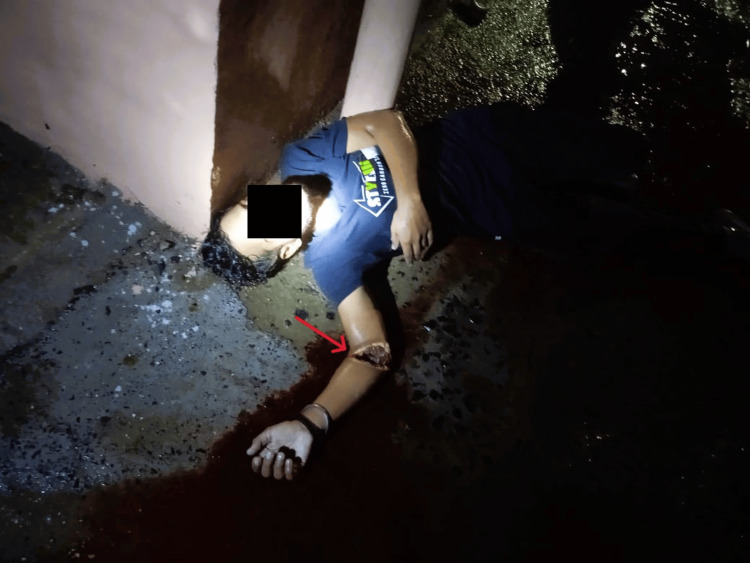
Body found roadside in pool of blood with incised wound at right elbow The image was provided by the police as part of the investigation.

A 20-year-old male person was found dead at the roadside. His body was in the pool of blood. Police noticed a large wound at his right elbow, and hence the body was brought for postmortem examination at our institute. The police inquest stated that the case seemed to be of homicide, and relatives of the deceased were also claiming the same (Figure 1).

Autopsy findings

**Figure 2:**
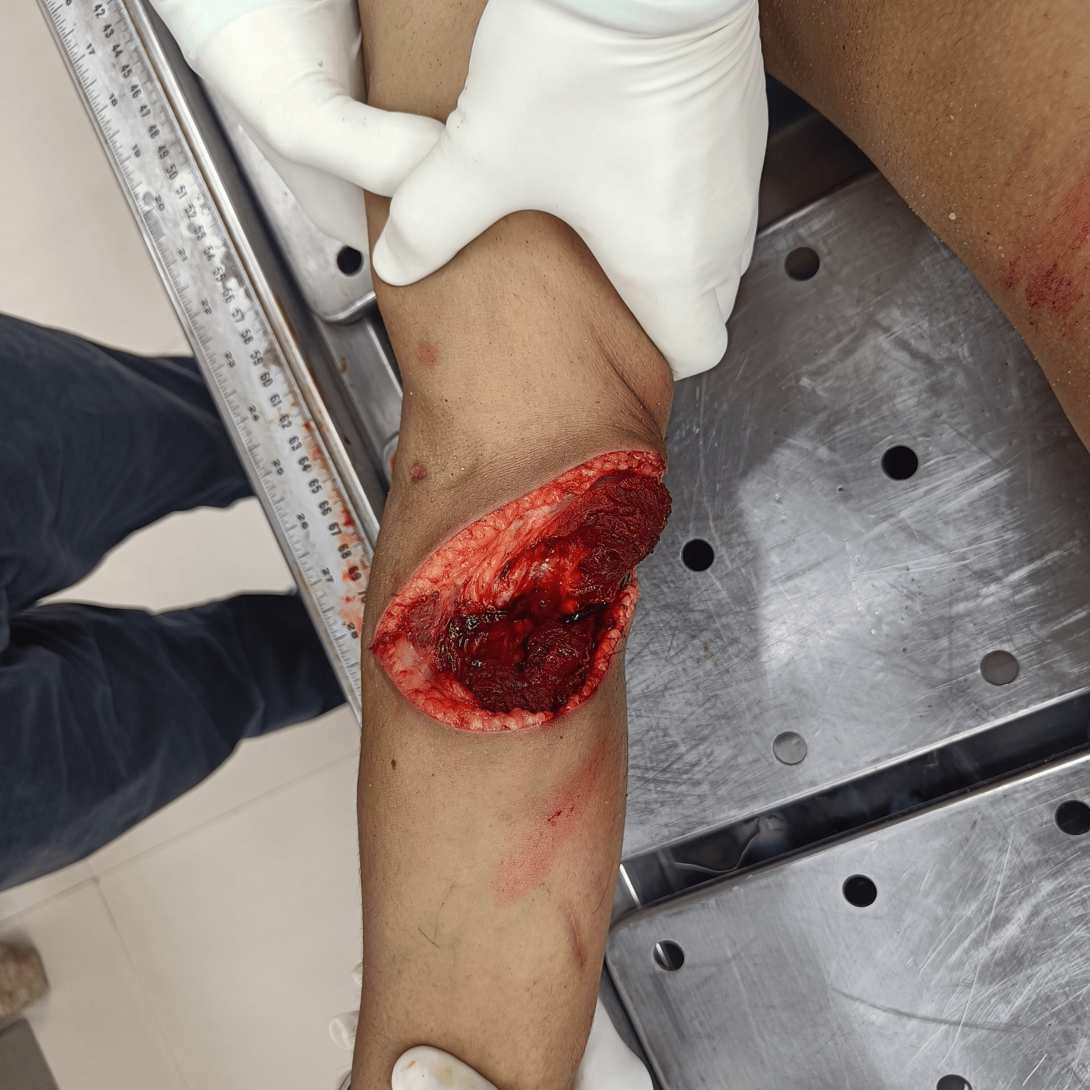
Wide gaping incised wound with sharp margins

An autopsy examination of the deceased reveals the presence of a wide, gaping, incised wound of size 13 cm x 6 cm x bone deep on the flexor aspect of the right forearm. The wound was obliquely placed; its medial end was at the right cubital fossa, and the lateral end was 4 cm below the right cubital fossa. The margins of the wound are sharp and clean-cut, and no tissue bridging was evident (Figure 2).

**Figure 3:**
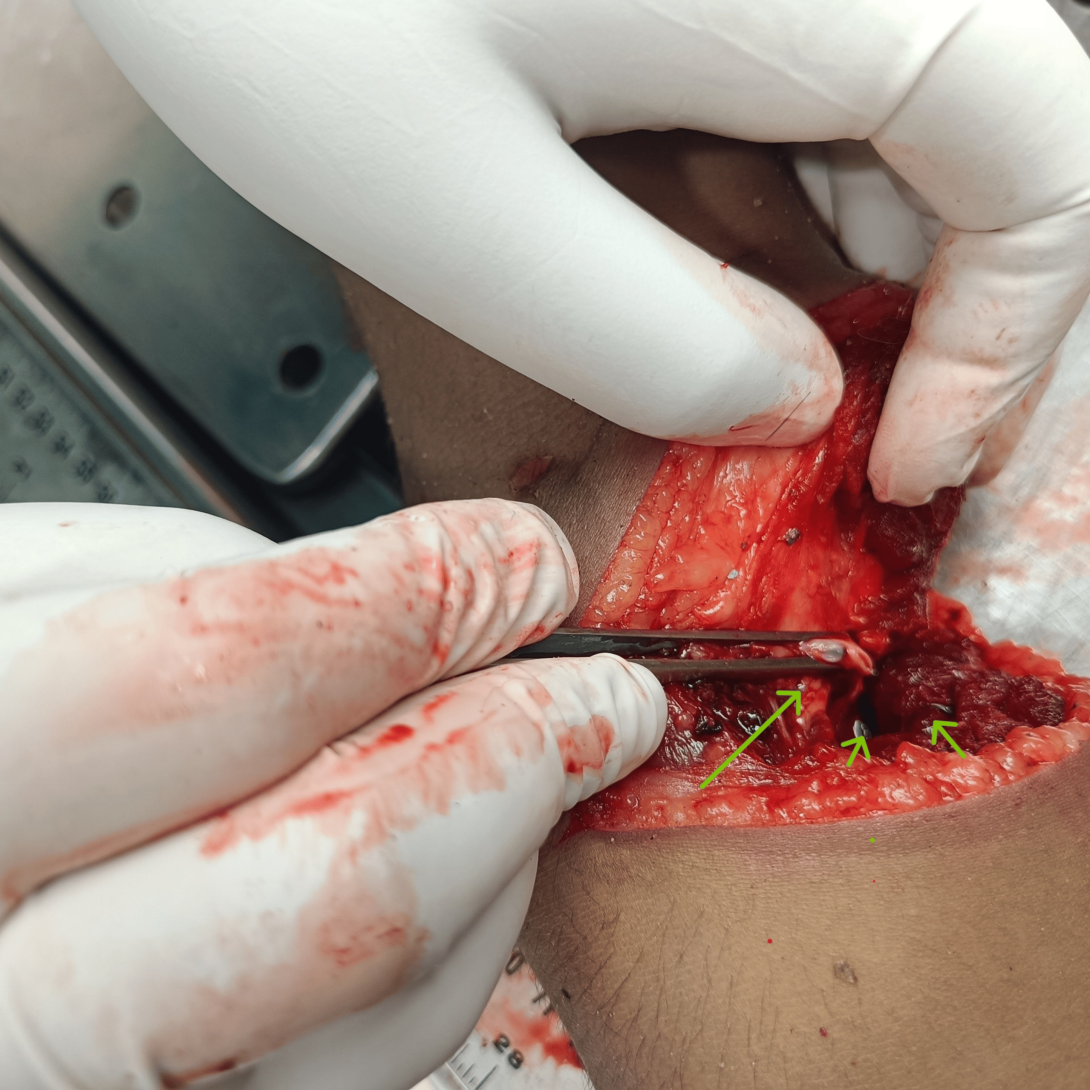
Wound with transected brachial artery at bifurcation (big arrow) with glass particles embedded (small arrows)

The underlying muscle at the wound was cut; both the ulnar and radial arteries after the bifurcation of the brachial artery were completely transected. A few small glass particles of about 1 mm in size were found embedded in the wound (Figure 3).

**Figure 4:**
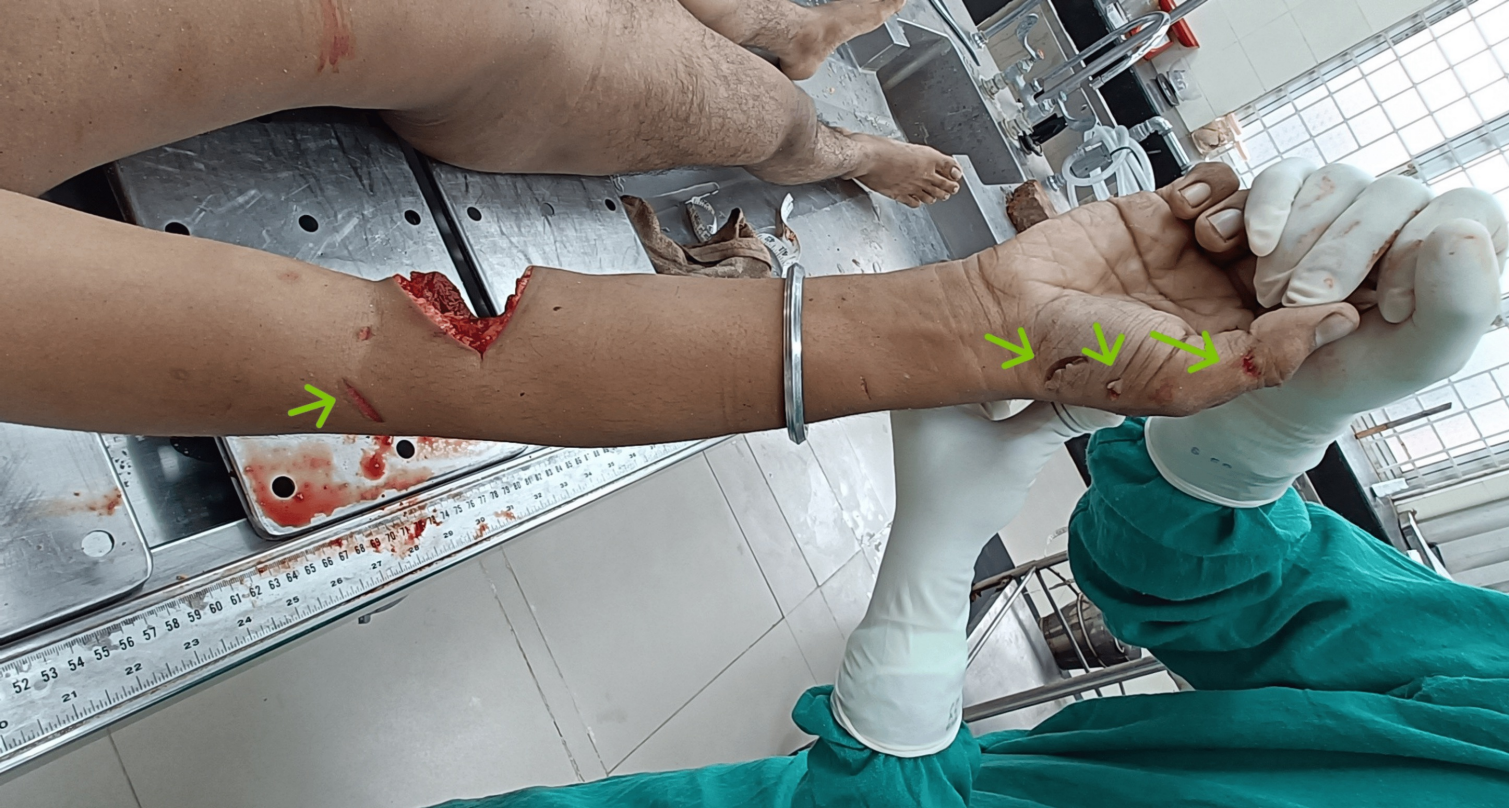
Small sharp cuts at palm

There were also two minor cut wounds noted surrounding the main fatal wound. Each of them was subcutaneously deep and 2 cm and 0.5 cm in length, respectively. Apart from this, three cut wounds were present at the base of the right thumb, and one cut wound was noted at the base of the right index finger on the palmar aspect of the right hand. No hesitation cut was noted. No injury on other body parts was noted (Figure 4). Postmortem examination revealed the cause of death due to hemorrhagic shock as a result of a sharp cut injury at the right cubital fossa. Postmortem examination also reveals an alcoholic odor (fruity odor) from the stomach for which viscera were preserved for chemical analysis.

Crime scene and circumstances

The crime scene involved a body lying at the corner of a road, surrounded by blood on the roadside. Initially, the police suspected it was a case of homicide due to a noticeably large cut on the deceased's hand and the body's location. The body was transported to our institute, where the deceased was declared dead, and an autopsy was performed.

**Figure 5:**
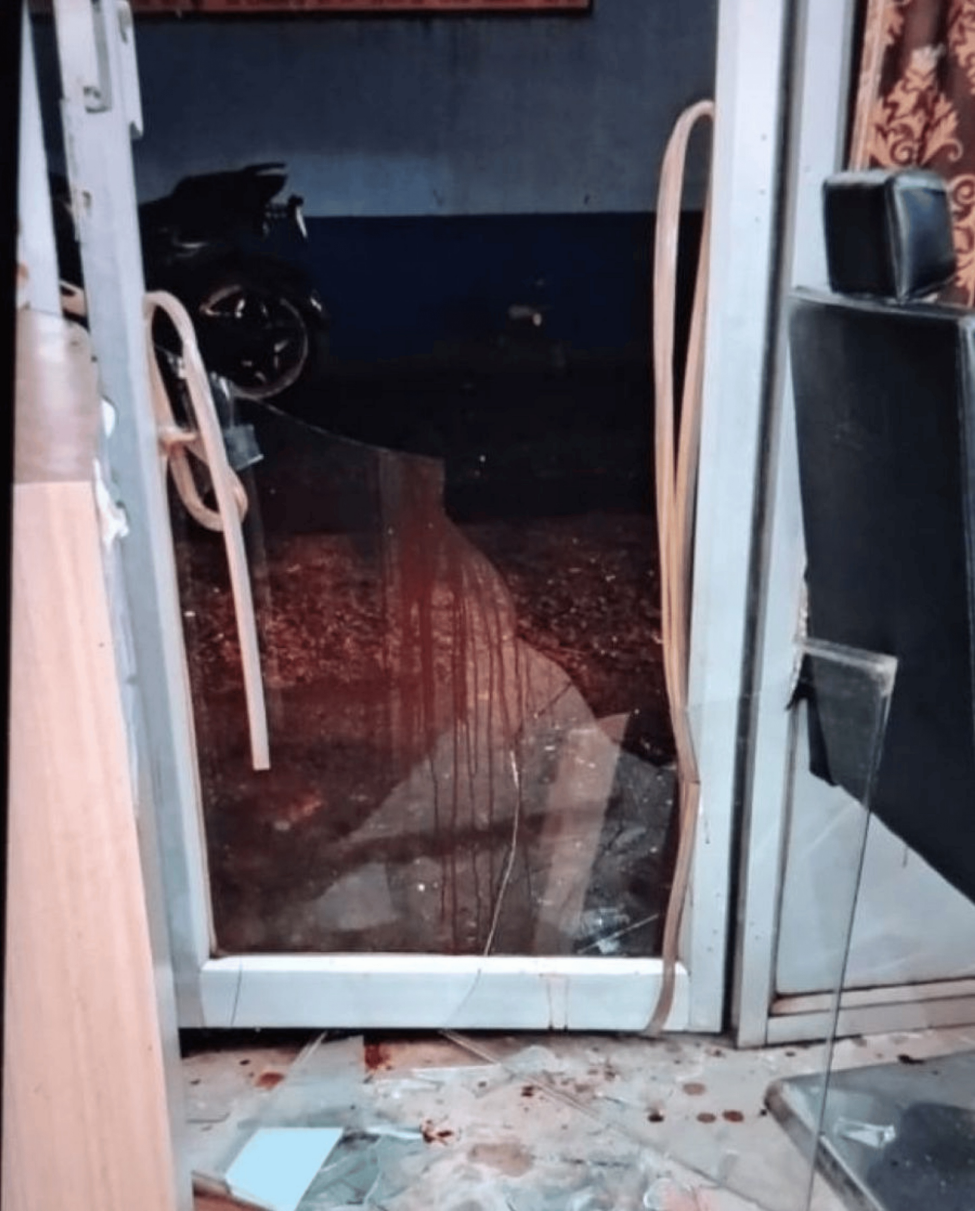
Broken glass door of shop with blood stains The image was provided by the police as part of the investigation.

During the investigation, the police discovered a shop located a few meters from the spot where the body was lying. The shop had a broken glass door with shattered glass particles scattered on the floor and bloodstains present on the door and surrounding area (Figure 5).

**Figure 6:**
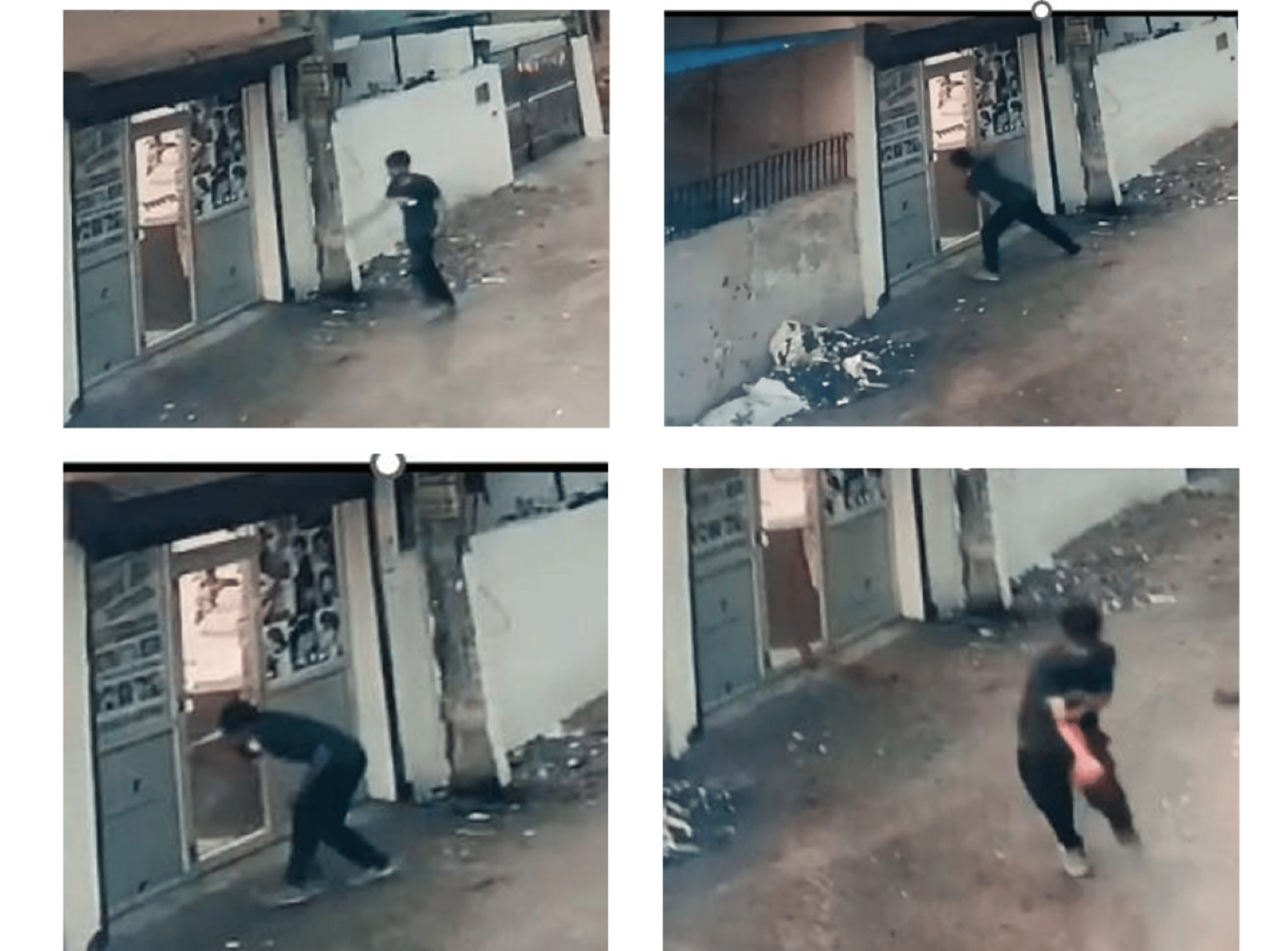
CCTV footage showing deceased hitting glass door of shop The image was provided by the police as part of the investigation.

The following day, the police obtained CCTV footage from a nearby installed camera. The footage revealed that the deceased had been wandering the road at night. It was a rainy night with little to no crowd on the road. The footage showed that the deceased appeared unsteady, resembling someone under the influence of alcohol. When he saw the glass door of the shop, he suddenly hit it with his right hand, causing the glass to shatter and inflicting a severe cut wound on his hand. He then ran away with the bleeding injury but collapsed a few meters away on the road due to excessive bleeding. Due to the rain, much of the blood on the road was washed away. However, bloodstains remained at the spot where the body was found, as well as on the floor of the shop and on the shop's door (Figure 6).

## Discussion

The accidental fatality in this case, which was initially misunderstood as homicide by the investigating authority, underscores the challenges in determining the manner of death when relying solely on injury morphology and preliminary scene assessment. This discussion explains the confounding factors, the role of investigative methods, and the integration of autopsy findings along with forensic and circumstantial evidence to arrive at an accurate conclusion.

The deep, gaping, incised wound on the flexor aspect of the right elbow resembled a chop wound. This kind of wound is typically caused by heavy, sharp instruments like axes and is often associated with homicide due to their severity and common use in violent acts [7]. The sharp, clean margins and transection of the brachial artery bifurcation further suggested forceful intent. Additionally, minor palmar cuts on the right hand initially raised suspicion of defense wounds. Defense wounds result when a victim tries to defend himself from the attack of the assailant. Defense wounds can be abrasions, lacerations, contusions, or incised wounds classically located on the ulnar aspect of the forearm or palms in victims resisting attack [8]. The body’s location a few meters from the injury site, compounded by washed-away blood due to rain, complicated the scene reconstruction, heightening suspicions of foul play.

However, the autopsy also reveals the observation that there were no signs of struggle; the wound was on the non-vital area, which is not usually seen in the case of homicide. In homicidal injury, the wound usually occurs in vital areas such as the neck, chest, or head, and the wounds are multiple in number [7,9]. While the current case has a solitary fatal wound, that is also in a non-vital area. Similarly, in suicidal cases, wounds are characterized by tentative cuts; the wounds are superficial in nature and usually involve accessible areas [7,10]. However the current case shows a deep cut wound on the right cubital fossa, which is not accessible in a right-handed person, so this finding makes a case against the favor of a suicidal wound.

Minor cuts were found on the palm of the hand, which resembled the defense wound and may have resulted from the individual hitting his hand forcefully on the glass door, which is evident from CCTV footage. This act of the person shattered the glass of the door into multiple pieces, and some of the glass pieces may have caused minor injury to his palm.

Hence, the autopsy finding has a mixed picture in classifying the manner of death. However, the CCTV footage was pivotal in reclassifying the manner of death in the current case. It revealed the intoxicated victim striking a glass door, resulting in a fatal vascular injury. The absence of tentative cuts or signs of struggle on autopsy, along with the victim’s solitary movement on the road, supported an accidental cause. Bloodstains on the shattered door, the victim’s impact with the glass, and the subsequent bleeding injury on his arm, as captured in the footage, confirmed the injury mechanism. This case underscores the limitations of autopsy findings in isolation, and without video evidence, the death might have been misclassified. One critical question needs to be answered: Can the glass cause such a severe injury? To explain the possibility, we need to understand the various patterns of injury possible by the glass particle.

Glass injury patterns are described in varied patterns in various forensic literature. As per Aggrawal A, glass-related injuries present as incised but may show irregularities at the edges. They should not be confused with lacerated wounds. Findings of broken pieces in the wounds confirm glass injury [11]. As per Reddy KSN, the wound by glass is a lacerated wound and may resemble an incised wound or stab wound. It is also mentioned that if a wound is caused by a sharp pointed particle of glass, it will be like a stab wound; similarly, the finding of broken glass in the wounds confirms the causation of glass. [7]. As per Knight B. Glass, a spike may cause a stab wound. Broken glass utensils/sheets can provide edges that equal or exceed surgical scalpels in their cutting ability [4]. Hence, it is evident that glass, though a common material, can inflict a diverse array of injuries depending on the type of glass particle involved, the mechanism of injury, and the context of impact. The wounds caused by glass can be categorized as incised, lacerated, and stab wounds due to their variable morphology.

Regarding the context of the use of glass as a weapon, data from Britain suggests that between 3400 and 5400 offenses occur annually in which glass is used as a weapon. Glass is commonly used as a cutting or stabbing weapon, mostly in bar fights. Drinking glasses or beer glass bottles are smashed and used as weapons of offense [12,13]. 

Choi et al. have mentioned that most of the fatal sharp wounds are homicidal or suicidal and rarely accidental. Most accidental sharp force wounds are due to broken glass [3]. There have been reported cases of accidental fatality due to broken glass, including sharp injury. Mileva B has reported a case with an accidental cutthroat injury due to a glass particle [14]. As per a study conducted by B. Karger, it has been mentioned that sharp-cut injuries are rarely accidental. In this study, they evaluated a total of 799 autopsies of victims of sharp cut injuries, out of which only 18 cases, i.e., 2.3% of cases, were found to be accidental. However, out of these 18 accidental sharp force injuries, in most of the cases, i.e., in 15 cases, the causative factor was a sharp glass particle [5].

Considering these literary sources, it cannot be denied that the glass particles could have caused the injury mentioned in the present case. Additionally, the presence of a few small glass particles inside the wound strongly supports the possibility that glass inflicted the wound. However, the CCTV footage has eliminated all doubts regarding the actual cause of the injury. 

Circumstantial evidence from the crime scene and digital evidence is crucial for determining the manner of death. It is said that every individual activity leaves digital traces, which can be used in the detection of crime [15]. There are multiple instances where digital data along with autopsy findings are used to solve a crime case [16]. In the present case also, the inclusion of crime scene details and CCTV footage strengthens the case for an accidental rather than homicidal cause, highlighting the value of combining forensic pathology with investigative data.

## Conclusions

This case demonstrates how fatal glass injuries can mimic homicidal violence, stressing the need for meticulous integration of forensic, circumstantial, and investigative data. The case emphasizes the limitations of autopsy findings alone in determining the manner of death. One should not always rely on history. It emphasizes that while injury patterns provide critical clues, their interpretation must be strengthened by comprehensive evidence review. Effective communication and collaboration with investigative agencies are crucial. Circumstantial evidence from crime scenes and CCTV footage can significantly aid in resolving medico-legal dilemmas (digital forensics). Thus, it advocates for a multidisciplinary approach to crime investigations.
